# Levels of HIV-1 persistence on antiretroviral therapy are not associated with markers of inflammation or activation

**DOI:** 10.1371/journal.ppat.1006285

**Published:** 2017-04-20

**Authors:** Rajesh T. Gandhi, Deborah K. McMahon, Ronald J. Bosch, Christina M. Lalama, Joshua C. Cyktor, Bernard J. Macatangay, Charles R. Rinaldo, Sharon A. Riddler, Evelyn Hogg, Catherine Godfrey, Ann C. Collier, Joseph J. Eron, John W. Mellors, ACTG A5321 Team

**Affiliations:** 1 Department of Infectious Diseases, Massachusetts General Hospital and Ragon Institute, Boston, Massachusetts, United States of America; 2 Division of Infectious Diseases, Department of Medicine, University of Pittsburgh, Pittsburgh, Pennsylvania, United States of America; 3 Center for Biostatistics in AIDS Research, Harvard T.H. Chan School of Public Health, Boston, Massachusetts, United States of America; 4 Department of Infectious Diseases and Microbiology, Graduate School of Public Health, University of Pittsburgh, Pittsburgh, Pennsylvania, United States of America; 5 Social & Scientific Systems, Inc., Silver Spring, Maryland, United States of America; 6 Division of AIDS, National Institute of Allergy and Infectious Diseases, Bethesda, Maryland, United States of America; 7 Department of Medicine, University of Washington, Seattle, Washington, United States of America; 8 Department of Medicine, University of North Carolina, Chapel Hill, North Carolina, United States of America; Emory University, UNITED STATES

## Abstract

Antiretroviral therapy (ART) reduces levels of HIV-1 and immune activation but both can persist despite clinically effective ART. The relationships among pre-ART and on-ART levels of HIV-1 and activation are incompletely understood, in part because prior studies have been small or cross-sectional. To address these limitations, we evaluated measures of HIV-1 persistence, inflammation, T cell activation and T cell cycling in a longitudinal cohort of 101 participants who initiated ART and had well-documented sustained suppression of plasma viremia for a median of 7 years. During the first 4 years following ART initiation, HIV-1 DNA declined by 15-fold (93%) whereas cell-associated HIV-1 RNA (CA-RNA) fell 525-fold (>99%). Thereafter, HIV-1 DNA levels continued to decline slowly (5% per year) with a half-life of 13 years. Participants who had higher HIV-1 DNA and CA-RNA before starting treatment had higher levels while on ART, despite suppression of plasma viremia for many years. Markers of inflammation and T cell activation were associated with plasma HIV-1 RNA levels before ART was initiated but there were no consistent associations between these markers and HIV-1 DNA or CA-RNA during long-term ART, suggesting that HIV-1 persistence is not driving or driven by inflammation or activation. Higher levels of inflammation, T cell activation and cycling before ART were associated with higher levels during ART, indicating that immunologic events that occurred well before ART initiation had long-lasting effects despite sustained virologic suppression. These findings should stimulate studies of viral and host factors that affect virologic, inflammatory and immunologic set points prior to ART initiation and should inform the design of strategies to reduce HIV-1 reservoirs and dampen immune activation that persists despite ART.

## Author summary

HIV-infected individuals who are receiving antiretroviral therapy continue to have low but persistent amounts of virus in blood as well as high levels of immune activation. Elevated immune activation has been linked to medical complications, like heart disease. Whether activation is being driven by or driving HIV persistence is not known. To answer this question, we measured levels of HIV, inflammation and immune activation in 101 HIV-infected individuals before and during long-term antiretroviral therapy. We found that pre-treatment levels of HIV correlated with on-treatment measures of HIV persistence. HIV levels correlated with inflammation and activation before starting therapy but not during long-term treatment, suggesting that virus persistence is not driving or driven by immune activation or inflammation. Higher levels of activation and inflammation before therapy were associated with higher levels during treatment, indicating that immune events that occurred before treatment initiation had long-lasting effects despite sustained control of the virus. These findings should stimulate studies of genetic or viral factors that affect levels of virus, activation and inflammation prior to treatment, and should inform the design of strategies to reduce HIV persistence and dampen harmful immune activation and inflammation in people who are on long-term treatment.

## Introduction

During the first year of effective antiretroviral therapy (ART), there is a marked reduction (10,000 to 100,000-fold) in plasma HIV-1 RNA levels to below the limit of detection of commercial assays (<50 copies/mL)[1–3]. Despite many years of virologic suppression, however, replication-competent HIV-1 persists in long-lived cellular reservoirs[4–6] and, if therapy is stopped, virologic rebound almost always occurs[7]. Individuals on ART continue to have detectable HIV-1 DNA in circulating CD4+ T cells and many also have measurable HIV-1 RNA in cells and plasma using sensitive detection methods[8]. Given the intense interest in reducing HIV-1 reservoirs to achieve ART-free remission or cure, determining the factors that impact the size and transcriptional activity of the infected cell population is critically important[9]; these determinants, however, are incompletely understood.

In addition to harboring HIV-1 in a long-lived reservoir, individuals on ART have persistently elevated levels of inflammation and T cell activation[10; 11]. Many potential mechanisms for these abnormalities have been proposed [11]. One mechanism may be compromised intestinal integrity from loss of mucosal CD4+ T cells shortly after HIV-1 acquisition leading to increased intestinal permeability and translocation of intestinal microbes and microbial products that trigger inflammation and immune activation[12]. Chronic exposure to other antigens or pathogens, like cytomegalovirus, may also contribute to persistent activation [13]. Another postulated mechanism for abnormal levels of inflammation and activation despite ART is ongoing HIV-1 replication or expression[14]. Conversely, abnormal inflammation and immune activation may contribute to HIV-1 persistence by driving proliferation and activation of HIV-1-infected cells and by activating uninfected cells that can support low-level viral replication[15]. If inflammation or activation in individuals on ART drives or is being driven by HIV-1 replication or expression, one would expect there to be a correlation between measures of HIV-1 persistence and levels of inflammation and immune activation.

Some cross-sectional studies of patients on ART have reported correlations between HIV-1 DNA or RNA and measures of immune activation [16; 17]; whereas others have not [18–20]. Cross-sectional studies, however, may be confounded because they do not adjust for pre-therapy levels of HIV-1, inflammation or activation; for this reason, longitudinal studies with access to pre-therapy samples are needed. Previous longitudinal studies have been small (<15 participants) [21–23] or have not examined the relation between HIV-1 levels, inflammation and activation [24].

To address these limitations, we evaluated longitudinal changes in HIV-1 levels in relation to inflammation and T cell activation in a large longitudinal cohort of chronically-infected participants who had sustained suppression of plasma viremia with samples stored before and during long-term ART. This design allowed us to assess the relationship between pre-ART and on-ART levels of HIV-1, inflammation and activation, as well as associations among these measures, adjusting for pre-therapy values. To our knowledge, this study is the largest longitudinal evaluation of the decay in measures of HIV-1 persistence in relationship to inflammation and T cell activation. We find that participants with high levels of HIV-1, inflammation and activation before starting ART continue to have high levels while on treatment, despite many years of virologic suppression. HIV-1 RNA levels before starting ART correlate with activation but no consistent associations were found among measures of HIV-1 persistence and inflammation or immune activation during long-term ART, suggesting that these abnormalities are not driving or being driven by viral persistence. These findings suggest that the “die is cast” by virologic and immunologic events that occur prior to initiation of ART and that studies to evaluate genetic or viral factors that affect HIV-1 and immunologic set-points will be needed to inform strategies aimed at reducing HIV-1 reservoirs, inflammation and activation that persist despite ART.

## Methods

### Study population

We evaluated a longitudinal cohort of participants with chronic HIV-1 infection who initiated ART in AIDS Clinical Trials Group (ACTG) trials for treatment-naïve persons and had subsequent follow-up while continuing to receive ART (ACTG studies A5001 [25] and A5321). Participants in the current study had plasma HIV-1 RNA levels < 50 copies/mL by commercial assays starting at week 48 of ART and at all subsequent time points, no reported ART interruptions, and paired plasma and peripheral blood mononuclear cell (PBMC) samples available from pre-ART and during ART. We measured HIV-1 levels, T cell activation and inflammatory biomarkers on stored samples obtained before ART and at years 1, 4 and (if available) once during years 6–15 on treatment. Data were censored for one participant subsequent to use of an agent that could affect the HIV-1 reservoir (chemotherapeutic medication). One participant who was hepatitis C virus (HCV) RNA positive was excluded.

### Ethics statement

The institutional review boards at the authors’ institutions approved the study. All participants provided written informed consent for their participation in the study (only adult participants were enrolled).

### Virologic assays

Cell-associated HIV-1 DNA, cell-associated HIV-1 RNA (CA-RNA) and 2-long terminal repeat (2-LTR) circles were measured by quantitative PCR (qPCR) in PBMC samples using methods that have been previously published [20; 26]. Longitudinal PBMC samples from each participant were thawed and assayed in the same qPCR run. Plasma HIV-1 RNA by single-copy assay (SCA) was measured at year 4 of ART using methods that have been previously published [27]. Primers and probes used for qPCR of HIV-1 DNA, CA-RNA and plasma HIV-1 RNA were identical [26; 27]. HIV-1 DNA and CA-RNA values per million CD4+ T-cells were calculated by dividing the total HIV-1 DNA or CA-RNA copies/million PBMCs (normalized for CCR5 copies measured by qPCR as published [26]) by the CD4+ T-cell percentage (x 0.01) reported from the same specimen date or from a CD4+ T-cell percentage imputed using linear interpolation from specimen dates before and after the HIV-1 DNA or CA-RNA results.

### Immunologic assays

To evaluate levels of soluble biomarkers, frozen, longitudinal plasma samples were thawed and analyzed in batches that included all the samples for a participant. Plasma concentrations of interleukin (IL)-6, high-sensitivity CRP (hsCRP), soluble CD14 (sCD14), and soluble CD163 (sCD163) were quantified using enzyme-linked immunosorbent assay (ELISA) kits per manufacturer’s instructions (R&D, Minneapolis, MN). Duplicates of 20% of the samples were included in each ELISA plate. Results were analyzed using BioTek ELx800 ELISA reader and KCjunior software (version 1.10). Levels of T cell activation and cell-cycling in cryopreserved PBMC from each participant were determined in batch using multicolor flow cytometry. PBMC samples were thawed, washed, and stained with the following antibodies: Live/Dead Aqua (Invitrogen, Grand Island, NY), CD3 APC-H7, CD4 Alexa 488, CD8 V450, HLA-DR PE, and CD38 APC (all BD Biosciences, San Diego, CA). To evaluate cell cycling, samples were permeabilized (Permeabilizing Solution 2, BD Biosciences) and stained with Ki-67 PerCP-Cy5.5 (BD Biosciences). Cells were then fixed in 1% paraformaldehyde, and analyzed using BD LSR Fortessa (FACSDiva) within 24 hours of staining.

### Statistical analysis

Rank-based correlations (Spearman) were performed for analyses; partial correlations (also rank-based) generated adjusted correlations. Groups were compared using the Wilcoxon rank-sum or Kruskal-Wallis test. To ensure sample adequacy for the CA-RNA analyses, we excluded results in which an internal control housekeeping gene, IPO8 [28], failed to reach a predetermined level of mRNA recovery [26]. For HIV-1 DNA values <3 and CA-RNA values <19 copies/million CD4+ T cells (which were below the limits of assay detection based on the number of CD4+ T cells analyzed), results were imputed to 0.5 copies/million CD4+ T cells and analyzed as the lowest rank. For longitudinal plotting, CA-RNA values <19 were plotted as 18 copies/million CD4+ T cells. For 2-LTR circles, results were treated dichotomously (positive vs. negative; negative = below the detection limit) [20]. Within-participant (log_10_-transformed) HIV-1 DNA change/year between pre-ART to year 1 on ART, year 1 to 4 on ART, year 4 on ART to the date of the last collected sample on ART was estimated with participant-specific linear regression models. Changes over time were compared against the null hypothesis of no change using the Wilcoxon signed-rank test.

## Results

### Study population

The study population consisted of 101 HIV-1-infected treatment-naïve participants who initiated ART and had plasma HIV-1 RNA levels consistently <50 copies/mL as measured by commercial assays at all time points at and after week 48 of treatment. The characteristics of the population (summarized in Table 1) were notable for the following: median age at ART initiation, 39 years; 21% female, median pre-ART plasma HIV-1 RNA of 4.6 log_10_ copies/mL and median pre-therapy CD4+ T-cell count of 290/mm^3^. Regimens at time of ART initiation were non-nucleoside reverse transcriptase inhibitor (NNRTI)-based in 43%, protease inhibitor (PI)-based in 41% and integrase strand transfer inhibitor (INSTI)-based in 19% (all participants on an INSTI received raltegravir). At the last longitudinal time point, participants had been on ART for a median of 7 years (interquartile range (IQR), 4–8), and the median CD4+ T-cell count was 681/mm^3^.

**Table 1:** Study population.

**Age at initiation of ART, median (Q1-3), years**	39 (31, 47)
**Age at the last longitudinal time point, median (Q1-3), years**	48 (38–53)
**Sex–female**	21%
**Race/Ethnicity**:	
White Non-Hispanic	51 (50%)
Black Non-Hispanic	19 (19%)
Hispanic (Regardless of Race)	29 (29%)
American Indian, Alaskan Native	2 (2%)
**Pre-therapy plasma HIV-1 RNA, median (Q1-3), log10 copies/mL**	4.6 (4.3–4.9)
**Pre-therapy CD4+ T-cell count, median (Q1-3), cells/mm3**	290 (162–379)
**Pre-therapy CD4:CD8 cell ratio, median (Q1-3)**	0.3 (0.2–0.5)
**Antiretroviral Regimen (Initial/At time last sample collected)**	
NNRTI-based	43%/42%
PI-based	41%/36%
INSTI-based	19%/24%
**Years on therapy at time last sample collected, median (Q1-3)**	7 (4–8)
**CD4 count at time last sample collected, median (Q1-3), cells/mm3**	681 (518–840)

### Pre-ART correlations between HIV-1 levels and CD4+ T-cell count

**Fig 1:**
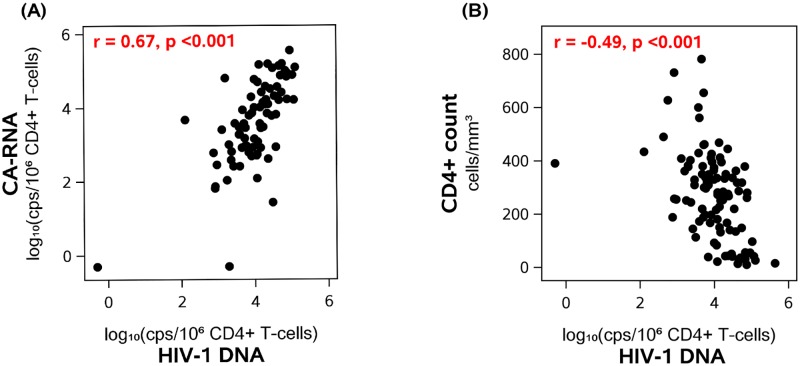
Correlations between pre-ART HIV-1 DNA and CA-RNA levels (A) and between pre-ART HIV-1 DNA and CD4+ T-cell counts (B).

Before initiation of ART, higher levels of plasma HIV-1 RNA were associated with higher levels of HIV-1 DNA (r = 0.47, p<0.001) and CA-RNA (r = 0.47, p<0.001). HIV-1 DNA and CA-RNA levels were also strongly correlated (r = 0.67, p<0.001) (Fig 1A); this correlation persisted after adjustment for pre-ART plasma HIV-1 RNA, indicating that the association was not simply related to higher plasma virus levels but rather that the level of transcription was directly related to the number of proviral templates. Participants with lower pre-therapy CD4+ T-cell counts had higher HIV-1 DNA levels (r = −0.49, p<0.001) (Fig 1B) and higher CA-RNA levels (r = −0.25, p = 0.027). Similarly, a lower pre-therapy CD4:CD8 cell ratio was associated with higher HIV-1 DNA (r = −0.46, p<0.001) and higher CA-RNA levels (r = −0.38, p<0.001).

Because plasma HIV-1 RNA has been reported to be higher in males than in females soon after HIV-1 acquisition [29], we compared pre-ART plasma HIV-1 RNA, HIV-1 DNA and CA-RNA levels between men and women. Median plasma HIV-1 RNA was higher in men than in women: 4.65 vs. 4.36 log_10_ copies/mL (p = 0.025), and this difference persisted even after adjusting for pre-ART CD4+ T-cell count. Similarly, median HIV-1 DNA, CA-RNA levels and the CA-RNA:DNA ratio were higher in men than in women (4.08 vs. 3.80 and 3.79 vs. 3.00 log_10_ copies/million CD4+ T-cells and 0.65 vs. 0.15, respectively), but these differences were not statistically significant (p = 0.08, 0.16 and 0.06 for the comparisons, respectively).

### Decline in HIV-1 DNA and cell-associated HIV-1 RNA after initiation of therapy

**Fig 2:**
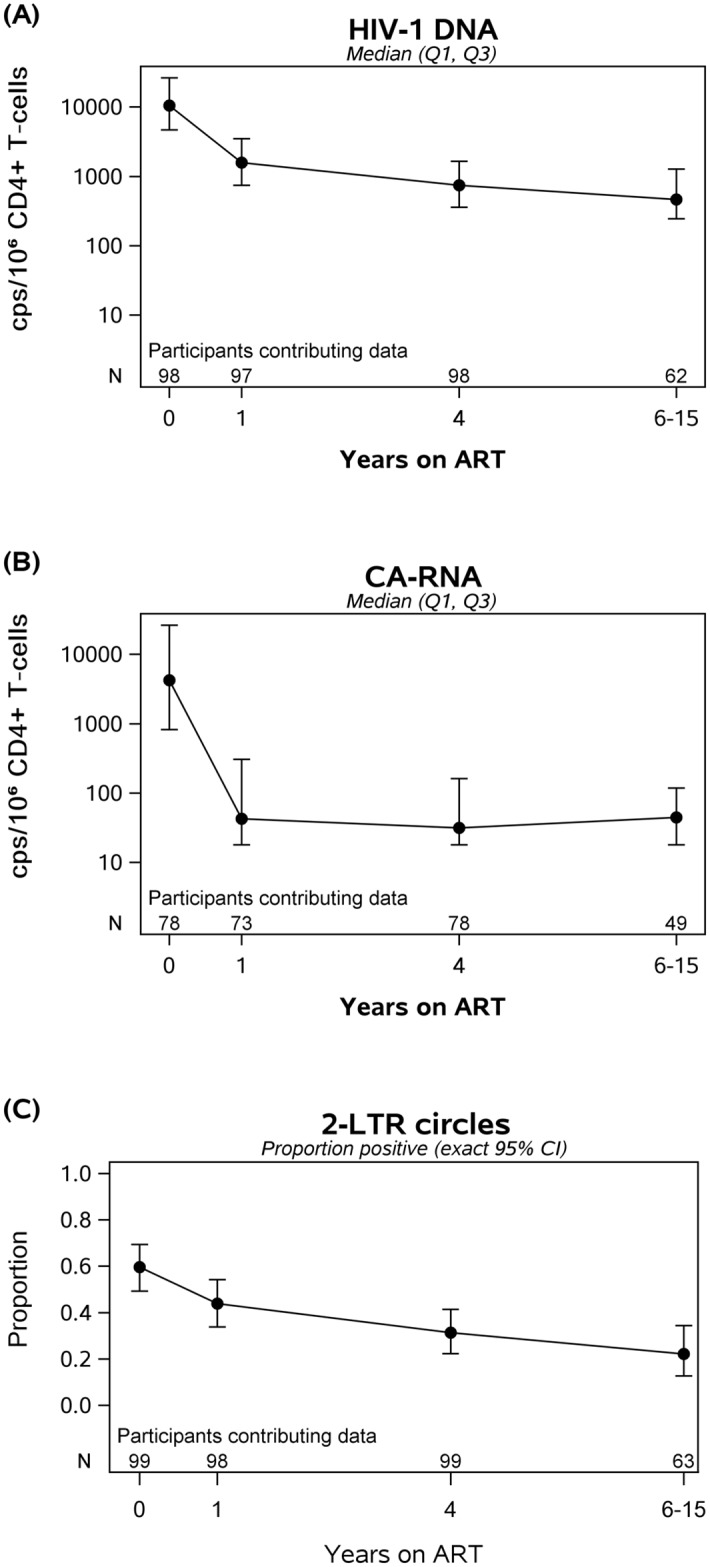
In participants on Antiretroviral Therapy (ART) with suppressed plasma viremia, longitudinal decay in HIV-1 DNA (A), cell-associated RNA (B) and proportion with detectable 2-LTR circles (C) over time. Between initiation of ART and year 4 of therapy, there was a 15-fold drop in HIV-1 DNA and a 525-fold drop in CA-RNA. After year 4 of ART, HIV-1 DNA decayed at 5% per year (half-life of 13 years); there was no further decay in CA-RNA levels.

After initiation of ART, all participants had a decrease in plasma HIV-1 RNA to <50 copies/mL by week 48 and continued to be below this threshold at all subsequent time points. We assessed HIV-1 DNA and CA-RNA levels at year 1, year 4 and (if available) at a later time point during year 6–15 of therapy. During the first 4 years of treatment, HIV-1 DNA declined by 15-fold (93% decline) whereas CA-RNA level fell 525-fold (>99% decline) (Fig 2A and 2B). The slope of HIV-1 DNA decay was greatest during the first 4 years of ART. However, even after 4 years of therapy, HIV-1 DNA levels continued to decline slowly (5% per year; p = 0.002) with an half-life of 13 years (95% confidence interval: 9, 56 years); 69% of participants had a negative slope. By contrast, there was no further decline in CA-RNA after year 1 of ART. There was no evidence that HIV-1 DNA or CA-RNA levels differed by initial ART regimen (at year 1, p = 0.12 and p = 0.15) (S1 Table). The percentage of participants who had detectable 2-LTR circles declined from 44% at year 1 of ART to 22% after at least 6 years of ART (Fig 2C); there was no evidence for an association between initial ART regimen and proportion of participants who were 2-LTR circle positive at year 1 (p = 0.26, S1 Table).

### Persistent correlations between pre-ART and on-ART virologic measures

**Fig 3:**
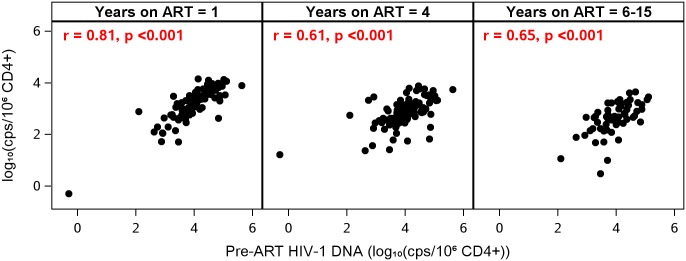
Pre-ART HIV-1 DNA Levels are correlated with on-ART HIV-1 DNA levels. Participants with high HIV-1 DNA before ART continued to have high HIV-1 DNA on therapy, despite sustained suppression of plasma viremia.

Despite suppression of plasma HIV-1 RNA to <50 copies/mL at all on-ART time points, participants with higher plasma HIV-1 RNA levels prior to ART continued to have higher levels of HIV-1 DNA after starting ART (e.g., at year 1, r = 0.38, p<0.001; at year 4, r = 0.26, p = 0.01). There were also strong correlations between pre- and on-ART HIV-1 DNA levels: participants who had higher HIV-1 DNA levels before ART continued to have higher DNA levels while receiving ART (at year 1, r = 0.81; at all other time points, r ≥ 0.61; all p-values <0.001) (Fig 3 and Table 2). Similarly, participants who had higher CA-RNA levels before ART had higher CA-RNA levels while receiving treatment (e.g., at year 1, r = 0.50, at year 4, r = 0.45, at year 6–15, r = 0.34, all p-values <0.02) (Table 2). HIV-1 DNA and CA-RNA, which were associated prior to starting ART (Fig 1A), continued to be correlated at all time points while on treatment (r = 0.52–0.59, all p<0.001).

**Table 2:** Correlations between pre-ART levels of HIV-1, inflammation, T cell activation and cycling and on-ART levels of HIV-1, inflammation, T cell activation and cycling.

Spearman correlation (p-value) with pre-ART level	1 year on ART	4 years on ART	6–15 years on ART
**HIV-1 DNA**	0.81 (<0.001)	0.61 (<0.001)	0.65 (<0.001)
**CA-RNA**	0.50 (<0.001)	0.45 (<0.001)	0.34 (0.017)
**IL-6**	0.41 (<0.001)	0.41 (<0.001)	0.39 (0.001)
**hs-CRP**	0.33 (<0.001)	0.48 (<0.001)	0.30 (0.015)
**sCD14**	0.40 (<0.001)	0.47 (<0.001)	0.45 (<0.001)
**sCD163**	0.53 (<0.001)	0.55 (<0.001)	0.58 (<0.001)
**%CD38+HLA-DR+ (CD4 cells)**	0.45 (<0.001)	0.23 (0.024)	0.18 (0.17)
**%CD38+HLA-DR+ (CD8 cells)**	0.33 (<0.001)	0.11 (0.29)	0.14 (0.27)
**%Ki67 on CD4+ T-cells**	0.45 (<0.001)	0.42 (<0.001)	0.38 (0.002)
**%Ki67 on CD8+ T cells**	0.49 (<0.001)	0.30 (0.003)	0.40 (0.001)

At year 4 of ART, we measured plasma HIV-1 RNA using an assay that detected as little as 0.4 copies/mL of virus (single-copy assay, or SCA). Fifty-seven percent of the participants had values below the limit of detection. We found that HIV-1 DNA correlated with plasma HIV-1 by SCA (r = 0.32, p = 0.01). However, CA-RNA did not correlate with plasma HIV-1 RNA by SCA (r = 0.03, p = 0.84), suggesting that most transcriptional activity was not resulting in detectable virion production.

At years 6–15 of ART, CA-RNA levels were significantly higher in men (n = 39) than in women (n = 10): median 68 vs. <19 copies/million CD4+ T cells, p = 0.005. However, there was no difference in HIV-1 DNA between sexes (471 vs. 463 copies/million CD4+ T-cells). The association between CA-RNA and sex was only seen at years 6–15 of ART and not at other time points.

### Immune deficiency associated with persistently high HIV-1 levels on ART

We assessed whether the correlation between low CD4+ T-cell counts and high HIV-1 DNA levels at time of ART initiation (Fig 1B) persisted after treatment was started. Lower CD4+ T-cell counts before starting ART were associated with higher HIV-1 DNA levels at all on-ART time points (r = −0.43 at year 1, −0.26 at year 4, −0.31 at years 6–15, all p-values <0.02). Similar associations were seen between lower pre-ART CD4:CD8 ratios and higher HIV-1 DNA levels while on therapy. We also examined whether impaired CD4+ T-cell recovery was associated with higher HIV-1 levels once participants were receiving ART. Lower on-therapy CD4:CD8 ratios were associated with persistently higher HIV-1 DNA at all on-ART time points (years 1, 4, 6–15; e.g., r = −0.49 at year 1 of ART, p<0.001).

### Measures of inflammation before starting ART

Because IL-6, hsCRP, sCD14 and sCD163 have been linked to mortality or end-organ disease in HIV-1-infected and uninfected populations [30], [31], we evaluated the pre-ART levels of these markers. There were modest associations between older age and higher hsCRP (r = 0.20, p = 0.042) and sCD14 (r = 0.22, p = 0.025). Lower pre-ART CD4:CD8 ratio was correlated with higher levels of several biomarkers: IL-6 (r = −0.28, p = 0.004); hsCRP (r = −0.22, p = 0.025); sCD14 (r = −0.17, p = 0.09); sCD163 (r = −0.19, p = 0.06). There was no evidence for an association between inflammatory biomarkers and sex.

When we assessed the pre-ART relationship between inflammation and HIV-1 levels, we found that higher pre-treatment plasma HIV-1 RNA was modestly correlated with higher sCD14 (r = 0.2, p = 0.042) and higher sCD163 (r = 0.19, p = 0.05). There were no associations between HIV-1 DNA and the inflammatory biomarkers. Similarly, CA-RNA was not correlated with IL-6, sCD14 and sCD163 but there was a modest correlation between hsCRP and CA-RNA (r = 0.27, p = 0.017 in an analysis adjusted for pre-ART plasma HIV-1 RNA).

### Change in inflammatory markers after starting ART

**Fig 4:**
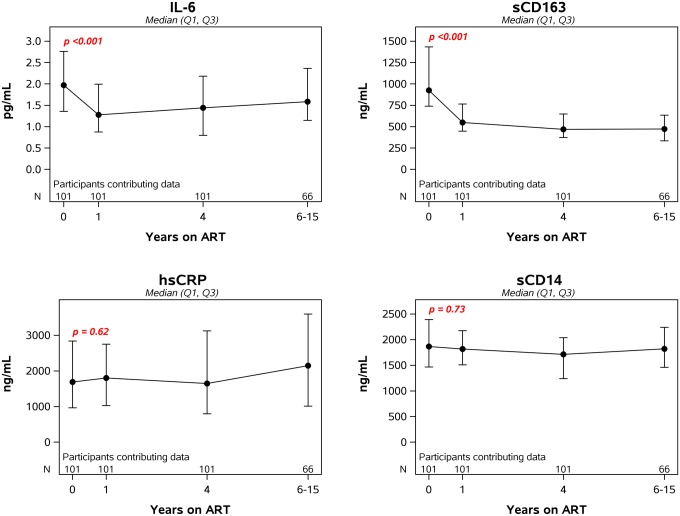
Longitudinal changes in markers of inflammation after initiation of antiretroviral therapy. The p-value in each panel is for the change in the log-transformed level of the specified biomarker from pre-ART to year 1.

Following initiation of ART, levels of IL-6 and sCD163 dropped significantly during the first year (34% and 46%, respectively) and then stabilized. By contrast, sCD14 and hsCRP did not decline after ART (Fig 4). Motivated by findings showing difference by initial ART class [32], we examined year 1 inflammatory markers by ART class. Soluble CD14 levels significantly differed by ART (NNRTI vs. PI vs. INSTI-based regimens, p = 0.003) with lower levels in those initiating INSTI-based regimens (S1 Table) which is consistent with the report by Hileman et al [32].

**Fig 5:**
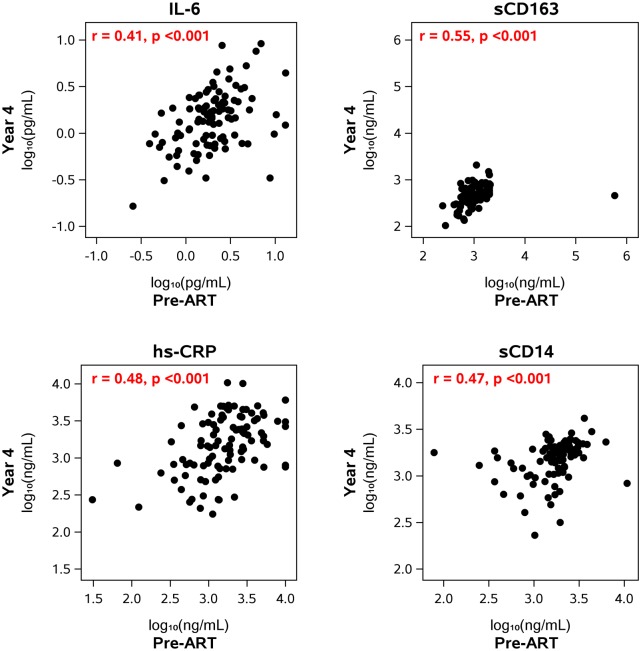
Correlation between pre- and on-ART levels of inflammatory biomarkers. The figures show the correlation between the levels of the biomarkers before starting ART and the levels at year 4 after starting therapy. The levels at the other on-ART time points (year 1, years 6–15) also correlated with the levels before starting ART (Table 2).

When we assessed the relationship between pre- and on-treatment levels of inflammation, we found pre-ART levels of IL-6, sCD163, sCD14 and hsCRP were significantly correlated with on-therapy levels of the same biomarkers at years 1, 4 and 6–15 of ART (Table 2). As examples, the correlation between pre-ART and year 4 on-ART sCD163 levels was 0.55 (p<0.001) and the correlation between pre-ART and year 4 on-ART sCD14 level was 0.47 (p<0.001) (Fig 5). The positive correlations between pre- and on-ART levels of all four biomarkers remained significant even after adjusting for both pre-ART plasma HIV-1 RNA and pre-ART CD4+ T-cell count.

Unlike the correlation seen between inflammatory biomarkers and CD4:CD8 ratio before starting treatment, once participants were receiving ART there was no correlation between the on-ART CD4:CD8 ratio (or CD4+ T-cell count) and IL-6, CRP or sCD14. At year 4 of ART, a lower CD4:CD8 ratio was associated with a higher sCD163 (r = −0.21, p = 0.03); however, this correlation was not seen at other time points.

### Inflammatory markers and virologic measures during ART

When we examined the relationship between measures of HIV-1 persistence and inflammation during ART, there were no consistent correlations between HIV-1 DNA or CA-RNA and the inflammatory biomarkers. For example, at year 4 of ART, there was no correlation between HIV-1 DNA and IL-6 (r = −0.07, p = 0.52), hsCRP (r = 0.1, p = 0.31), sCD14 (r = −0.13, p = 0.22) or sCD163 (r = 0.07, p = 0.46). At year 4 of ART, we also assessed the relationship between plasma HIV-1 RNA by SCA and inflammation: there was no association between plasma HIV-1 RNA and any of the inflammatory biomarkers, e.g., for IL-6, r = 0.02, p = 0.87.

### T cell activation before starting ART

We evaluated the pre-treatment levels of T cell activation measured by co-expression of CD38 and HLA-DR. Prior to initiation of ART, the median percentage of activated CD4+ T-cells was 15.1 (IQR, 9.5–21), and the median percentage of activated CD8+ T-cells was 43.7 (IQR, 34.8–59.1).

CD4+ and CD8+ T-cell activation were strongly correlated with each other before ART was initiated (r = 0.66, p<0.001). Pre-treatment plasma HIV-1 RNA level was also significantly associated with both CD4+ and CD8+ T-cell activation (r = 0.41 and 0.47, respectively, both p values <0.001). Lower CD4+ T-cell count was associated with higher CD4+ T-cell activation (r = −0.34, p<0.001) and higher CD8+ T-cell activation (r = −0.33, p<0.001). The correlations between lower CD4:CD8 ratio and higher activation were even stronger (r = −0.46 for CD4+ T-cell activation, r = −0.44 for CD8+ T-cell activation, p <0.001).

### T cell activation during antiretroviral therapy

**Fig 6:**
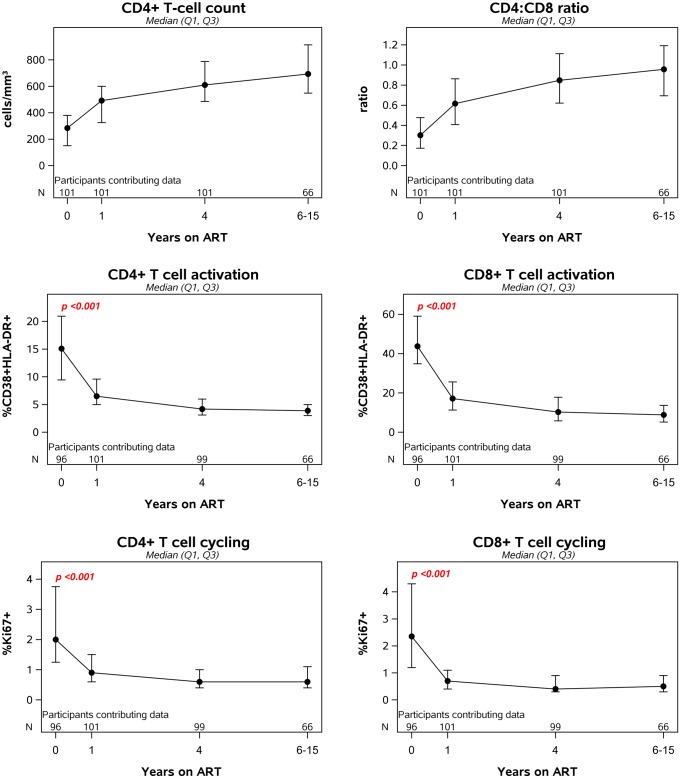
Longitudinal changes in T cell counts, ratios, activation and cycling after initiation of antiretroviral therapy.

Following initiation of ART, there was a significant increase in CD4+ T-cell count and CD4:CD8 ratio (Fig 6). CD4+ and CD8+ T-cell activation dropped significantly during the first year after initiation of ART (49% and 57% decline, respectively). There was no evidence that CD4+ or CD8+ T-cell activation differed by initial ART regimen when examined at year 1 (p = 0.46 and p = 0.95) (S1 Table). Participants with high levels of cellular activation before starting treatment continued to have high levels while on ART although this correlation diminished over time on ART (Table 2). For example, pre-ART CD4+ T-cell activation was correlated with on-ART CD4+ T-cell activation at year 1 (r = 0.45, p<0.001) and year 4 (r = 0.23, p = 0.02); the significant association at year 1 (but not at year 4) persisted after adjustment for pre-ART plasma HIV-1 RNA and pre-ART CD4+ T-cell count. After 6–15 years of ART, a significant association was no longer detected between pre-ART and on-ART CD4+T-cell activation (Table 2). Similarly, pre-therapy CD8+ T cell activation was associated with on-therapy CD8+ T cell activation at year 1 of ART but not at later time points.

Incomplete CD4+ T-cell reconstitution while on ART was also associated with elevated immune activation. We examined whether on-ART CD4+ T-cell count or CD4:CD8 ratio is associated with on-ART CD4+ T-cell activation. We found that lower CD4+ T-cell count and lower CD4:CD8 ratio at year 1 and 4 of ART were associated with higher CD4+ T-cell activation at the corresponding time point, which is consistent with a prior report [33].

### T cell activation and HIV-1 levels before and during ART

We examined the relationship between levels of HIV-1 persistence and immune activation before therapy was initiated. As noted above, pre-ART plasma HIV-1 RNA level was significantly associated with both CD4+ and CD8+ T-cell activation (r = 0.41 and 0.47, respectively, p<0.001). There was also a correlation between pre-ART HIV-1 DNA levels and T cell activation (r = 0.27 and 0.29 for CD4+ and CD8+ T-cells, respectively, p<0.01), but these associations were no longer evident when adjusted for pre-ART plasma HIV-1 RNA. By contrast, correlations between pre-ART CA-RNA and immune activation (for CD4 cells, r = 0.37, p = 0.001; for CD8 cells, r = 0.4, p<0.001) persisted after adjustment for pre-ART plasma HIV-1 RNA.

**Fig 7:**
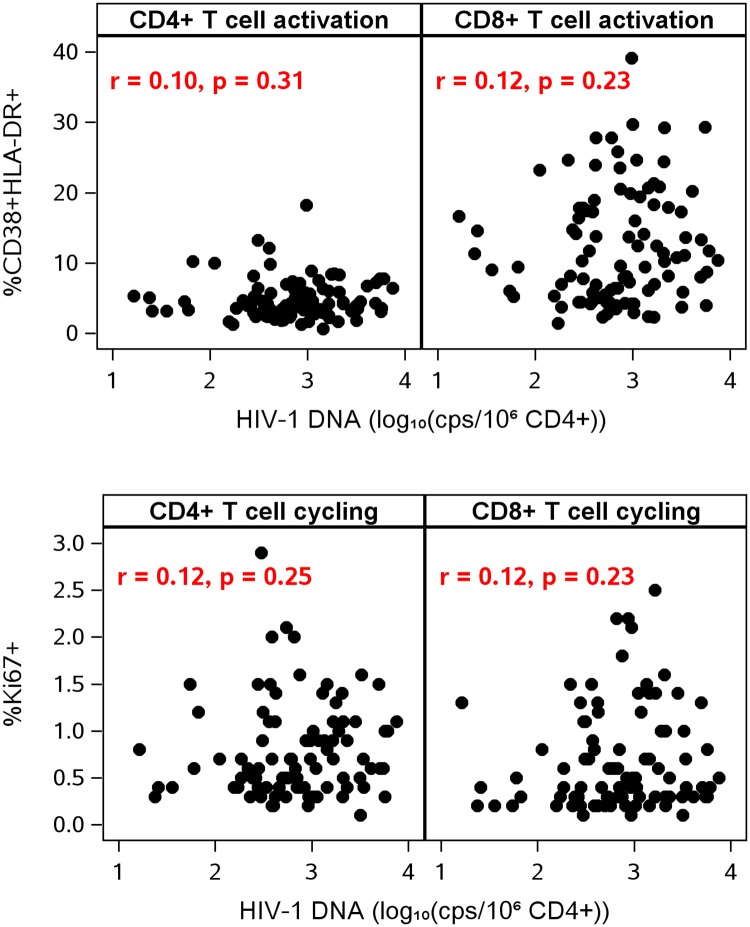
No association between HIV-1 DNA and T cell activation or T cell cycling. Data in this figure are from year 4 of antiretroviral therapy.

After initiation of ART, there was little evidence of associations among measures of HIV-1 persistence and levels of T-cell activation at each on-treatment time point. At year 1 of ART, only the association of HIV-1 DNA with CD4+ T-cell activation was significant after adjustment for pre-ART HIV-1 RNA (r = 0.22, p = 0.032). At year 4 of ART and thereafter, there were no longer any significant associations between T cell activation and HIV-1 DNA or CA-RNA (Fig 7): for example, CD4 activation and HIV-1 DNA at year 4 (n = 96): r = 0.10, p = 0.31; at years 6–15 (n = 62): r = −0.06, p = 0.65). Similarly, there was no association between T cell activation and plasma HIV-1 RNA by SCA at year 4. This lack of association was not because of lower power to detect correlations; at year 4, the number of participants contributing data to the HIV-1 DNA analysis (n = 96), for example, was similar to that at the pre-ART or year 1 time points (n = 93 and n = 97, respectively). We also did not find evidence for correlations between HIV-1 DNA or CA-RNA and CD4+ or CD8+ T cell activation in analyses stratified by sex.

### T-cell cycling

Prior to initiation of ART, the median percentage of cycling Ki-67+ CD4+ T-cells was 2.0 (IQR, 1.3–3.8) and the median percentage of cycling Ki-67+ CD8+ T-cells was 2.3 (1.2–4.3). CD4+ and CD8+ T-cell cycling were strongly associated with each other (r = 0.7, p<0.001). Higher pre-ART CD4+ T-cell cycling was also associated with higher pre-treatment HIV-1 RNA (r = 0.23, p = 0.026) and lower CD4:CD8 ratio (r = −0.30, p = 0.003). We also found evidence for pre-ART correlations between T-cell cycling and T-cell activation. There was a strong correlation between CD4+ T-cell cycling and CD4+ T-cell activation (r = 0.59, p<0.001), that persisted after adjustment for plasma HIV-1 RNA level, and between CD4+ T-cell cycling and CD8+ T-cell activation (r = 0.34, p<0.001). CD8+ T-cell cycling was associated with CD4+ and CD8+ T-cell activation (r = 0.33 for both comparisons, p = 0.001), but not with CD4:CD8 ratio or CD4 cell count. We did not find evidence for associations between CD4+ or CD8+ T-cell cycling and soluble markers of inflammation.

Following initiation of ART, CD4+ and CD8+ T-cell cycling declined significantly (Fig 6): during the first year of therapy, there was a 48% and 60% decline, respectively. As had been seen for inflammation and activation, participants with high levels of T-cell cycling before starting treatment continued to have high levels while on ART (Table 2). For example, the correlation between pre-ART CD4 T cell cycling levels and year 4 levels was 0.42 (p<0.001). These correlations remained significant even after adjustment for both pre-ART HIV-1 RNA and pre-ART CD4+ T- cell count.

Finally, we analyzed the relationship between T-cell cycling and cellular measures of HIV-1. Prior to ART, CD4+ and CD8+ T-cell cycling were not significantly associated with HIV-1 DNA or CA-RNA. During ART, there were sporadic associations between T-cell cycling and cellular HIV-1 DNA and RNA levels but these were not significant after adjustment for pre-ART plasma HIV-1 RNA. At year 4 of ART, there was no correlation between HIV-1 DNA and CD4+ or CD8+ T-cell cycling (both r = 0.12, both p-values ≥0.23) (Fig 7); there was no correlation between CA-RNA and CD4+ or CD8+ T cell cycling (r = 0.02 and 0.00, respectively; both p-values ≥0.89); and there was no correlation between plasma HIV-1 RNA by SCA and CD4+ or CD8+ T-cell cycling (r = −0.20 and −0.16, respectively; both p-values ≥0.12).

## Discussion

To address the limitations of previous studies in assessing the determinants of the size and decay of HIV-infected cell populations, we evaluated longitudinal changes in markers of HIV-1 persistence in relation to inflammation, T-cell activation and cycling in a large cohort of persons who initiated ART and had sustained suppression of plasma viremia for many years. The greatest decline in HIV-1 DNA levels occurred during the first 4 years of ART but there was a continued slow decline even after that time point (5% per year). Individuals with higher HIV-1 DNA and CA-RNA levels before starting ART continued to have higher levels while on treatment, indicating that once a large population of infected cells is established, it remains large despite many years of suppressive ART. Although inflammation and activation were associated with plasma HIV-1 RNA before treatment was initiated, we did not find correlations between measures of HIV-1 persistence and levels of inflammation or activation once individuals were on long-term ART.

One of the most contentious debates in current HIV-1 research is whether ongoing viral replication is driving the abnormally elevated levels of inflammation and immune activation in individuals on long-term antiretroviral therapy[34; 35]. There is little doubt that HIV-1 replication can induce inflammation and immune activation. Indeed, we did find in the current study that, prior to initiation of ART, plasma HIV-1 RNA levels and immune deficiency correlate with inflammation and T cell activation. If ongoing viral replication (or virus production) is also inducing inflammation and activation in treated individuals, one might reasonably expect that measures of the infected cell population, like HIV-1 DNA, cell-associated HIV-1 RNA or plasma HIV-1 RNA, would correlate with levels of inflammatory biomarkers and T cell activation. The fact that we observe no such correlations in individuals who are on long-term ART casts doubt on the concept that ongoing viral replication (or virus production) is responsible for elevated levels of inflammation and activation once plasma viremia is consistently suppressed by ART.

A second concept that has been proposed is that chronic inflammation and immune activation are key drivers of the persistence of HIV-1 reservoirs during ART[15]. If this were the case, a direct correlation would be expected between levels of HIV-1 persistence and inflammation, immune activation or T cell cycling on ART. Our analysis did not identify any consistent correlations between virologic and immunologic markers after ART, which is contrary to the proposed concept. Rather, the strongest associations found for markers of HIV-1 persistence (plasma HIV-1 RNA, HIV-1 DNA and CA-RNA) on ART were the pre-ART levels of each virologic measure.

What then is responsible for abnormal levels of inflammation and activation in individuals on antiretroviral therapy? While this study cannot answer this question, it may provide clues as to the cause. One of the most important observations of this longitudinal study is that high levels of inflammation, immune activation and T cell cycling before treatment correlate with high levels during long-term therapy, despite many years of sustained virologic suppression. This finding suggests that, for inflammation, activation and T cell turnover in HIV-infected persons, the “die is cast” by pathogenic mechanisms that occur well before therapy is initiated. Mechanisms consistent with our observations are those that predominate during untreated infection but can exert a long-lasting effect, such as fibrotic damage to lymphatic tissue [36], increased intestinal permeability leading to elevated microbial translocation, or persistent co-infections in the setting of compromised immune surveillance. The important implication of our findings is that strategies designed to reverse the damage induced prior to ART initiation, rather than interventions aimed at reducing HIV-1 persistence, will be needed to ameliorate the inflammation and activation that contribute to end-organ diseases despite ART. Our findings should stimulate studies of viral and host factors that affect levels of inflammation, activation and T cell turnover prior to starting ART.

Our study also sheds light on the factors that influence the size of the infected cell population in persons on ART. We observed that HIV-1 DNA declines to a lesser extent than CA-RNA (15-fold vs. 525-fold by year 4). The more rapid and extensive decline in CA-RNA than HIV-1 DNA suggests that the subset of cells expressing HIV-1 RNA has a shorter half-life than infected cells that do not express RNA. The shorter half-life of HIV-1 RNA-expressing cells could be explained either by translation of viral proteins that result in immune recognition and clearance of the cells or death of the cells from viral protein-mediated cytopathicity. The additional finding that cellular HIV-1 RNA does not appear to decline after year 1 of ART suggests that there is longer-term persistence of a subset of cells expressing hypermutant or defective HIV-1 transcripts that do not result in cell death or clearance at a rate higher than their proliferation. These observations are consistent with recent reports that cells harboring defective HIV-1 proviruses can express unusual RNA transcripts[37].

Because of the size and duration of our longitudinal study, we were also able to detect a slow but significant decline in HIV-1 DNA levels even after four years of suppressive ART (5% per year, estimated half-life 13 years). This half-life is similar to the recently reported decay of plasma HIV-1 RNA (measured by single copy assay) that continues after 4–11 years of ART (half-life 11.5 years [3]) but is longer than estimates of the half-life for cells with inducible, replication-competent virus (3.7 years; 95% CI 2.3–9.5 years) [38; 39]. These findings indicate that HIV-1-infected cell populations, including those producing virions, do not persist indefinitely and that effort should be directed toward accelerating the inherent slow decay of such cells. Finally, the observations that high levels of HIV-1 DNA many years after initiation of therapy are linked to high pre-treatment levels of HIV-1 DNA and RNA and to immune deficiency and incomplete CD4+ T-cell recovery strongly support early initiation of ART to reduce the size of the infected cell population that persists on ART. Recent data from trials of early ART initiation [40–42] are consistent with this concept.

Some of our results contrast with those from a large (n = 190) cross-sectional study that showed a modest correlation between CA-RNA levels and CD4+ T-cell activation in participants on ART [16]. In that study, participants had been suppressed on ART for a median of 31 months, or a little over 2.5 years, compared with 7 years at the last time point in the current study. The shorter duration of ART in the previous study may explain the different results if HIV-1 and activation levels were still declining and had yet not reached a steady state. Indeed, after one year of ART, we find that HIV-1 DNA is modestly correlated with CD4+ T-cell activation (r = 0.22, p = 0.032); however, at year 4 of ART and thereafter, there were no longer significant associations between T cell activation and HIV-1 DNA (Fig 7) or CA-RNA. Another potential difference between the studies is the degree of virologic suppression: participants in the previous study were allowed to have viral blips to 1000 copies/mL and some had plasma HIV-1 RNA levels between 50 and 150 copies/mL whereas all participants in the current study had documented sustained virologic suppression <50 copies/mL. Finally, in the previous study 25% of the participants had a CD4+ T-cell count <250/mm^3^ at the time of the cross-sectional analysis whereas in this study <1% had a count below that threshold at the last longitudinal time point. We note, however, that many participants in the current study had low pre-ART CD4+ T-cell counts (median 290/mm^3^ with 25% of participants <162/mm^3^) and that the higher CD4+ T-cell counts at the last time point are a result, at least in part, of durable long-term suppression.

This study has some limitations. Our assessment of HIV-1 persistence relied on nucleic-acid based measurements. In particular, HIV-1 DNA overestimates the size of the replication-competent reservoir [43]. In addition, the demonstration that defective proviruses may be transcribed[37] raises a question as to whether cell-associated HIV-1 RNA is a reliable measure of the size of the HIV-1 reservoir. Another study, however, found that higher cell-associated HIV-1 RNA levels predict shorter time to virus rebound when ART is stopped [42], suggesting CA-RNA does reflect the size of the HIV-1 reservoir. Prospective trials to define biomarkers that correlate with the size of the replication competent HIV-1 reservoir are needed to settle the question. A second limitation is that we measured activation and proliferation on total CD4+ and CD8+ T cell populations; specific cellular subsets may be important for HIV persistence[44; 45] and additional studies of these sub-populations are warranted. Another limitation is that our study focused on HIV-1 persistence in blood but a significant portion of the total body HIV-1 reservoir is in tissues [46]; because tissue HIV-1 levels may not be adequately reflected by blood measurements, additional studies of tissue reservoirs are needed. Finally, participants in this study were selected to have sustained virologic suppression while on long-term ART. Other HIV-infected individuals, who are less adherent with ART, may have different results; for example, suboptimal adherence has been shown to be associated with elevated inflammation [47], highlighting that even brief medication lapses have a measurable impact.

The longitudinal design of this study also confers several important strengths. The participants in this cohort had sustained virologic suppression below the limits of detection of commercial assays, mitigating the potential effects of virologic failure or transient viremia on measures of HIV-1 persistence, inflammation or immune activation that might be observed in less-adherent individuals. Because this study was able to measure levels of HIV-1, inflammation, immune activation and T cell turnover in samples taken before ART was initiated as well as during long-term treatment, we were able to adjust for pre-ART values to address the confounding that may occur in cross-sectional studies. Another strength of our study is that its longitudinal design allows us to control for duration of ART when examining the relationship between levels of HIV-1, inflammation and activation in participants with documented sustained virologic suppression. Because HIV-1 levels, inflammation and activation decline after starting ART, it is possible that the apparent association between HIV-1 persistence and activation in some prior cross-sectional studies may have been confounded by duration of ART; our study, in which we control for duration of ART, avoids this important limitation of prior studies. The large size of the cohort also allowed us to detect slow but continuous decay of HIV-1 DNA and to assess longitudinal associations between inflammation, activation and viral persistence with greater certainty than in previous studies. Finally, our study shows that inflammation, activation and cycling decline after initiation of ART to a relatively stable level. The stability of these levels in individuals who have been on long-term ART suggests that interventions designed to reduce these abnormalities could be first assessed in single-arm trials before being evaluated in randomized trials.

Taken together, the data from this large longitudinal study provide strong evidence that the pre-treatment numbers of infected cells and levels of inflammation and activation are the key determinants of their persistence during ART and cast doubt on the concepts that inflammation and activation are driven by HIV-1 replication or drive HIV-1 persistence in individuals on ART. We anticipate that these and other findings from this study will inform future interventions designed to reduce HIV-1 reservoirs and persistent inflammation and immune activation in individuals on long-term ART.

## Supporting information

S1 Table Association between Year 1 measures of HIV-1 persistence, inflammation and T cell activation with initial Antiretroviral (ARV) regimen. (DOCX)
