# Supplementary material for: Levels of HIV-1 persistence on antiretroviral therapy are not associated with markers of inflammation or activation
Source: PLoS Pathog. 2017 Apr 20;13(4):e1006285. doi: 10.1371/journal.ppat.1006285 (PMC5398724; doi:10.1371/journal.ppat.1006285)
Supplement: S1 Table — (DOCX) [file ppat.1006285.s001.docx]

**Supplementary Table: Association between Year 1 Measures of HIV-1 Persistence, Inflammation and T cell Activation with Initial Antiretroviral (ARV) Regimen.**

|  | | **Initial ARV Regimen** | | |  |
| --- | --- | --- | --- | --- | --- |
|  | | **NNRTI + NRTIs (N=41)** | **PI + NRTIs (N=39)** | **INSTI + other (N=19)** | **P-Value*** |
| Pre-ART plasma HIV-1 RNA (log₁₀cps/mL) | N | 41 | 39 | 19 | 0.531 |
|  | Median | 4.61 | 4.53 | 4.67 |  |
|  | Q1, Q3 | 4.39, 4.87 | 4.17, 4.92 | 4.32, 5.07 |  |
|  | | | | | |
| HIV-1 DNA (log₁₀(cps/10⁶ CD4+)) at year 1 on ART | N | 39 | 37 | 19 | 0.119 |
|  | Median | 3.30 | 3.12 | 3.52 |  |
|  | Q1, Q3 | 2.76, 3.53 | 2.67, 3.49 | 3.04, 3.87 |  |
|  | | | | | |
| HIV-1 DNA (log₁₀(cps/10⁶ CD4+)) slope/yr: yrs 0-1 | N | 39 | 37 | 19 | 0.364 |
|  | Median | -0.86 | -0.89 | -0.93 |  |
|  | Q1, Q3 | -1.02, -0.60 | -1.07, -0.62 | -1.19, -0.78 |  |
|  | | | | | |
| CA-RNA (log₁₀(cps/10⁶ CD4+)) at year 1 on ART | N | 27 | 32 | 14 | 0.145 |
|  | Median | 1.55 | 1.40 | 2.17 |  |
|  | Q1, Q3 | 1.40, 2.39 | -0.30, 2.52 | 1.62, 2.65 |  |
| 2-LTR circles at year 1 on ART | Negative | 20 (51%) | 20 (53%) | 14 (74%) | 0.261** |
|  | Positive | 19 (49%) | 18 (47%) | 5 (26%) |  |
| IL-6 (pg/mL) at year 1 on ART | N | 41 | 39 | 19 | 0.095* |
|  | Median | 1.29 | 1.42 | 1.03 |  |
|  | Q1, Q3 | 0.89, 2.03 | 0.95, 2.07 | 0.67, 1.33 |  |
|  | | | | | |
| hs-CRP (ng/mL) at year 1 on ART | N | 41 | 39 | 19 | 0.179 |
|  | Median | 2,040 | 1,356 | 1,026 |  |
|  | Q1, Q3 | 1,453, 2,642 | 882, 2,719 | 459, 4,111 |  |
|  | | | | | |
| sCD14 (ng/mL) at year 1 on ART | N | 41 | 39 | 19 | 0.003 |
|  | Median | 1,887 | 1,932 | 1,378 |  |
|  | Q1, Q3 | 1,592, 2,337 | 1,611, 2,194 | 971, 1,785 |  |
|  | | | | | |
| sCD163 (ng/mL) at year 1 on ART | N | 41 | 39 | 19 | 0.536 |
|  | Median | 594 | 495 | 549 |  |
|  | Q1, Q3 | 474, 785 | 429, 740 | 455, 764 |  |
|  | | | | | |
| %CD38+HLA-DR+ on CD4+ cells at year 1 on ART | N | 41 | 39 | 19 | 0.459 |
|  | Median | 7 | 6 | 7 |  |
|  | Q1, Q3 | 6, 9 | 4, 9 | 5, 10 |  |
|  | | | | | |
| %CD38+HLA-DR+ on CD8+ cells at year 1 on ART | N | 41 | 39 | 19 | 0.946 |
|  | Median | 16 | 18 | 18 |  |
|  | Q1, Q3 | 11, 27 | 11, 25 | 11, 26 |  |
|  | | | | | |

Non-nucleoside reverse transcriptase inhibitor (NNRTI); nucleoside reverse transcriptase inhibitor (NRTI); Protease inhibitor (PI); Integrase strand transfer inhibitor (INSTI).

*Kruskal-Wallis Test
**Fisher's Exact Test
